# *Planctomycetes* as a Vital Constituent of the Microbial Communities Inhabiting Different Layers of the Meromictic Lake Sælenvannet (Norway)

**DOI:** 10.3390/microorganisms8081150

**Published:** 2020-07-29

**Authors:** Julia E. Storesund, Anders Lanzèn, Eva-Lena Nordmann, Hilde Rief Armo, Olga Maria Lage, Lise Øvreås

**Affiliations:** 1 Institute of Marine Research, 5817 Bergen, Norway; julia.storesund@hi.no; 2 AZTI, Marine Research Division, Pasaia, Spain and IKERBASQUE—Basque Foundation for Science, E-4813 Bilbao, Spain; alanzen@azti.es; 3 Department of Biological Sciences, University of Bergen, 5020 Bergen, Norway; Eva-Lena.nordmann@uib.no (E.-L.N.); hilde.armo@uib.no (H.R.A.); 4 Department of Biology, Faculty of Sciences, University of Porto, 4169-007 Porto, Portugal; olga.lage@fc.up.pt; 5 Interdisciplinary Centre of Marine and Environmental Research, University of Porto, 4450-208 Porto, Portugal

**Keywords:** bacteria, diversity, meromictic lake, *Planctomycetes*, cultivation

## Abstract

Meromictic lakes are permanently stratified lakes that display steep gradients in salinity, oxygen and sulphur compounds tightly linked to bacterial community structure and diversity. Lake Sælenvannet is a meromictic lake located south of Bergen, Norway. The 26 m deep lake is connected to the open sea and permanently stratified into two layers separated by a chemocline. The upper water layer is brackish with major input from water runoff from the surroundings. The bottom layer consists of old saline water with low or no oxygen concentrations. Bacteria from phylum *Planctomycetes* are reported to be ubiquitous in lake environments. They are involved in the degradation of complex carbon sources in aquatic environments and are also linked to anaerobic processes such as fermentation and sulphur reduction. To study *Planctomycete* distribution along a chemical gradient, we sampled the water column throughout Lake Sælenvannet in 2012 and profiled the microbial community using 16S rRNA amplicon sequencing (metabarcoding) with 454 pyrosequencing. *Planctomycetes* related 16S rRNA gene sequences were found to be present both in the oxic and anoxic parts of the lake and showed an uneven distribution throughout the water column, with the highest relative abundance of 10% found in the saline anoxic layer at 15 m depth. In a follow-up study in 2014, samples from eight different depths were collected for enrichment and isolation of novel *Planctomycetes*. This study resulted in successful isolation in pure culture of 10 isolates affiliated to four different genera from the family Planctomycetaceae. One strain closely related to *Blastopirellula cremea* was isolated from 9 m depth, and two novel strains affiliated to the genera *Stieleria* and *Gimesia* were isolated at 7 and 9 m depths, respectively. Furthermore, seven isolates with identical 16S rRNA gene sequences were retrieved from seven different depths which varied greatly in salinity and chemical composition. These isolates likely represent a new species affiliated to *Rubinisphaera*. The adaptation of this novel *Planctomycete* to water depths spanning the entire chemical gradient could indicate a high phenotypic plasticity and/or a very efficient survival strategy. Overall, our results show the presence of a diverse group of *Planctomycetes* in Lake Sælenvannet, with a strong potential for novel adaptations to chemical stress factors.

## 1. Introduction

Lakes are fundamental parts of life linked to the structure and benefit of society and natural ecosystems. These ecosystems represent important reservoirs for plants, animals and microbes, and any changes in their environmental quality and water renewal rates can have wide-ranging ecological implications. Changes in air temperature, precipitation and catchment area, together with depth, have direct effects on the physical, chemical and biological characteristics of lakes. A typical lake has distinct zones of biological communities, closely linked to its physical structures. Lakes are stratified into three separate sections: the epilimnion, the metalimnion and the hypolimnion. The epilimnion or surface layer is the top-most layer in a thermal or chemically stratified lake, occurring above the metalimnion and the deeper hypolimnion. It is warmer and typically has higher dissolved oxygen concentration than the hypolimnion. The metalimnion is the water layer in a stratified lake which lies beneath the epilimnion and above the hypolimnion, in which the temperature and/or oxygen decreases rapidly with depth. 

Meromictic lakes are lakes that are permanently stratified, as deeper water masses are not renewed during seasonal circulation. This is usually due to enrichment of soluble minerals in the deep water, such as bicarbonate, calcium, magnesium, iron and manganese. These accumulations of salt and minerals can be due to bacterial activities and processes in the deep water and in sediments, or from mineral sources in the area. The deep waters containing salt enrichments are referred to as monimolimnion, whereas the uppermost circulating water masses in a meromictic lake are referred to as mixolimnion. The transition zone between the two layers are termed the chemocline. The enrichment of salts in the monimolimnion results in water masses with a high mass density. To mix these heavier water masses with the lower density mixolimnion, additional energy is required.

Bacteria play a prominent role in lake ecosystems and greatly impact lake water quality, but the bacterial taxa participating in these activities remain largely undescribed [1]. Globally, the most predominant freshwater bacterioplankton from the epilimnion are Actinobacteria, Proteobacteria (Beta- and Alphaproteobacteria), and *Bacteroidetes* [1,2]. However, the understudied hypolimnion is teeming with microbes that have not yet been cultured. Recently, oxygenated hypolimnion from 10 deep freshwater lakes with a variety of geochemical characteristics were investigated, and the bacterioplankton community composition was described [3]. The study identified several predominant lineages inhabiting multiple lakes and found ubiquity and quantitative significance of bacterioplankton lineages in the oxygenated hypolimnion of these lakes. They also found that the most abundant hypolimnion-specific lineages were Chloroflexi, *Planctomycetes* and Marine Group I Thaumarchaeota, which exhibited abundances ranging from 1.5–32% of the bacterioplankton community [3]. 

*Planctomycetes* are an enigmatic group of bacteria with large genomes that follow complex lifestyles and display unusual cell biological features. Although *Planctomycetes* have been isolated from a variety of environments, including aquatic, marine and freshwater ecosystems, host-associated systems, sediment, soil and artificial systems, the total number of isolated *Planctomycetes* is still limited compared to other phyla [4]. Most of the presently isolated family Planctomycetaceae are considered (micro-) aerobic chemoorganotrophic microorganisms that are able to degrade complex organic compounds. In a recent study of lacustrine sediments, 60 *Planctomycete* metagenome-assembled genomes (MAGs) were assembled from 10 large metagenomic datasets [5]. These data, together with spatiotemporal abundance patterns (using CARD-FISH (catalyze reporter deposition fluorescence in situ hybridization)), were used to elucidate the evolutionary history of lacustrine *Planctomycetes* and their genome evolution patterns linked to their lifestyle strategies [5]. According to [6], *Planctomycete* abundance ranges from 0 to 11% of the aquatic planktonic prokaryotic community. Due to the relevance of *Planctomycetes* in nitrogen and carbon biogeochemical cycles [7,8,9,10,11], their potential role in aquatic ecosystem functioning is being recognized. The first evidence for *Planctomycetes* with anaerobic metabolism was reported by a microbial diversity study of an anaerobic digester of a municipal wastewater treatment plant that showed that activated sludge contained a highly diverse number of *Planctomycetes* that were able to grow under both aerobic and anaerobic conditions [12]. In a recent study from a boreal lake in Northern Russia, a new strain PX69^T^ was isolated that was affiliated with the *Pirellula*-related Pir4 clade, which is dominated by environmental sequences retrieved from a variety of low-oxygen habitats [13]. 

In this study, we focus on Lake Sælenvannet, which is a meromictic lake located south of Bergen, Norway (60° 20′ 4.8″ N, 5° 16′ 34.3″ E). It is permanently stratified in two layers, mixolimnion and monimolimnion. The bottom layer naturally contains old marine water with a higher salinity than the brackish upper water layer. The interface between the layers, the chemocline, is a hotspot for microbial activity. 

Previous studies from 1997 of Lake Sælenvannet identified the location of the chemocline to be at a 5 m depth and found that a distinct shift in community composition followed. The surface layer was inhabited by a high number of undescribed bacteria, a high abundance of *Chlorobium phaeovibrioides* was found in and around the chemocline, and the presence of sulphate reducers and methanogens was detected below the chemocline [14]. Since 1997, the chemocline of Lake Sælenvannet started to move upwards in the water column, and in the last years chemocline was found relatively near the surface, resulting in a severe sulphide smell with evident noxious consequences. To lower the chemocline and prevent the sulphide smell, the lake has been oxygenated from the bottom since 2010, which may have altered the bacterial community and distribution. 

In this study, two new sampling campaigns were carried out throughout the Lake Sælenvannet water column. In the 2012 campaign, six layers from all lake zones were analyzed for microbial community composition using 454 sequencing. As a considerable amount of *Planctomycetes* were detected in the mixolimnion and monimolimnion, a follow-up study was done in 2014, with the main focus to enrich and isolate candidates of the hitherto scarcely studied *Planctomycetes*. Much of the diversity of *Planctomycetes* is still not known, which hampers the understanding of the overall lake ecology. We hypothesize that the microbial community structure and the composition of the plankton community will vary depending on the lake chemical and physical parameters. The results will help to illuminate the association of *Planctomycetes* in lake system communities and the potential contribution of these bacteria in the biogeochemical cycles in lake systems. 

## 2. Materials and Methods

### 2.1. Sampling Site

Water samples were collected from the meromictic Lake Sælenvannet located south of Bergen, Norway. The lake contains old relict seawater from the surrounding fjords. It has a maximum depth of 26 m and consists of two main basins. The lake is referred to as a meromictic lake as there is a distinct chemocline separating the aerobic surface layer with freshwater input from the surroundings from the anaerobic old saltwater found at the bottom of the lake. The lake is connected to Nordåsvannet via a relatively narrow and shallow channel and then further connected to the fjord system and the open sea. Depending on the amount of freshwater runoff from land, precipitation and climatic variations, the location of the chemocline varies. Usually, it is located at a depth of 3 to 5 m.

### 2.2. Water Sampling

In May 2012, water samples were obtained from the mixolimnion (1 m depth), chemocline (5 m) and the monimolimnion (5.5, 6, 7 and 15 m depth) in Lake Sælenvannet. These samples were used for microbial community profiling. In October 2014, water samples were collected from 8 depths. Five samples were from the mixolimnion (1, 4, 7, 8 and 8.5 m depths), and 3 samples were from the monimolimnion (9, 10 and 15 m depths). These samples were used for enrichment, isolation and transmission electron microscopy (TEM) characterization. In both years, the samples from the upper 8.5 m were taken with a submersible pump, as described by [15], in which the water was pumped up from a defined depth through a tube with a manual vacuum pump. The samples from 9 m and below were taken with a Niskin bottle. One liter of water was sampled from each sampling depth at both sampling times. Temperature and salinity were measured with a salinoterm and a YSI model 33 SCT meter (Yellow Springs Instrument Co., Yellow Springs, OH, USA), and dissolved oxygen was measured with an oxygen electrode and a YSI model 57 oxygen meter (Yellow Springs Instruments, Yellow Springs, OH, USA). 

### 2.3. DNA Extraction from Environmental Samples and 454 Pyrosequencing

To collect microbial biomass for DNA analysis, water was filtered directly through 0.2 μm Sterivex filter units (Merck, Millipore, MA, USA) via a peristaltic pump without prefiltration, and stored immediately at −80 °C until further analysis. DNA was extracted using the CTAB (cetyl trimethylammonium bromide) method, as described by [16], with the exception that the phenol:chloroform:isoamylalcohol step was omitted. 

Amplicon pyrosequencing of the variable V6–V9 region of the 16S rRNA gene was conducted, as previously described by Bengtsson et al. [17], using primers 787F (5′-ATTAGATACCCNGGTAG-3′) and 1492R (5′-GNTACCTTGTTACGACTT-3′) [18]. The PCR was done in triplicate, and the reactions were pooled. The cleaned PCR products were used as a template for a second nested PCR with the same primers using unique multiplex identification tags (barcodes). The amplicons were then pooled in equimolar amounts and stored at −80 °C until they were processed for pyrosequencing at the Norwegian Sequencing Centre using GS-FLX Titanium technology (454 Life sciences, Roche, Branford, CT, USA) in Oslo, Norway. The raw sequences were processed, filtered and noise was removed from the pyrosequencing reads using AmpliconNoise (version 1.1, [19]), which corrected and compensated for errors introduced during PCR and pyrosequencing, thereby improving the estimation of bacterial diversity. Denoised sequences were then clustered into Operational Taxonomic Units (OTUs) using complete linkage clustering, as described by Quince et al. [19], with a similarity cutoff of 97%. To taxonomically classify the OTUs, their representative sequences were aligned to the SilvaMod database v128 (released 2017; https://github.com/lanzen/CREST) using blastn (v.2.6.0) + task megablast) and thereafter classified using CREST with default parameters [20]. Sequences unclassified at the domain level, likely not derived from rRNA genes, as well as mitochondrial or plastid 16S rRNA gene sequences, were removed from further analyses. Diversity indexes and rarefied richness estimates were calculated using the R package vegan [21] and relative taxon abundances were plotted using the R package ggplot2 [22]. The raw data (SFF files) were submitted to the European Nucleotide Archive with study accession number PRJEB39146.

### 2.4. Isolation of the Planctomycete Strains

To cultivate *Planctomycetes*, we used a two-step approach which involved an initial enrichment of the samples at 1:10 and 1:100 dilutions of lake water in M30 medium [23], followed by plating on solid M13 media [24]. The M30 medium contained (g per liter) *N*-acetylglucosamine, 2.0; Na_2_HPO_4_ × 2H_2_O, 0.01; 10× vitamin solution no. 6 [25], 1.0 mL; Hutner’s basal salts [26], 20 mL; 0.1 M Tris:HCL pH 7.5, 50 mL; aged seawater, 700 mL. The M13 medium contained (g per L) Bacto-peptone, 0.2; yeast extract, 0.2; glucose, 0.2; 10× vitamin solution no. 6, 1.0 mL; Hutner’s basal salts, 20 mL; 0.1 M Tris:HCL pH 7.5, 50 mL; ampicillin, 0.2; aged seawater, 700 mL; Gelrite, 5.0. As the majority of previously isolated heterotrophic *Planctomycetes* are resistant to several antibiotics [27,28,29], additional parallel enrichment cultures in M30 medium for each depth were supplemented with ampicillin (0.2 g/L) and streptomycin (0.2 g/L) to selectively target the *Planctomycetes*. Enrichments were initiated under both anaerobic and aerobic conditions and incubated at room temperature (~22 °C) with a natural light cycle of day and night. After 7 or 11 days of incubation, all enrichment cultures were diluted in M30 medium (1:10 and 1:100), and 200 μL aliquots were plated on solid M13 medium. Cultures were incubated at room temperature. The growth of potential *Planctomycetes* was visualized using light microscopy, and promising cultures were examined using transmission electron microscopy on negatively stained samples. Colonies with pigmentations towards red, pink and orange were regarded as promising, as well as smaller white round colonies. If these characteristics fell together with microscopy observations showing rosette formations, budding reproduction, dense cell aggregates and stalk formations, these were further processed. Favorable colonies were re-streaked three times on solid M13 medium to obtain pure cultures, and verified as *Planctomycetes* by sequencing the 16S rRNA gene using bacterial specific primers A8f and 1542r, as described by Storesund and Øvreås [30]. 

### 2.5. Electron Microscopy of Isolates

Electron micrographs of the communities from different depths in Lake Sælenvannet were prepared by placing a drop of native sample on top of copper grids (400 mesh Cu grids; Agar Scientific, Ltd., Essex, UK) supported with carbon-coated formvar films and let to air dry. Each grid was negatively stained with 2% uranyl acetate for 30 s, and the cells were inspected using a JEOL 100CX transmission electron microscope (JEOL Ltd., Akishima, Japan) operated at 80 kV.

## 3. Results

### 3.1. Lake Hydrography

**Figure 1:**
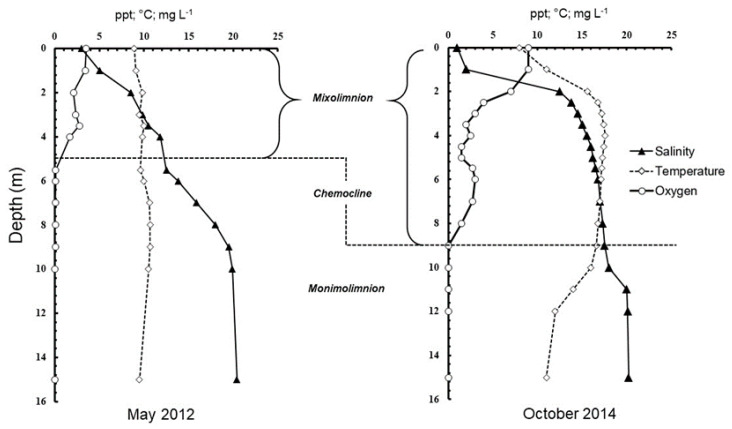
Hydrography profiles from Lake Sælenvannet in May 2012 and October 2014. In May 2012, the mixolimnion extended down to 5 m depth, and in October 2014, it extended down to 9-m depth. In 2012, the lake was anoxic below 5.5 m depth, whereas in 2014 it was only anoxic below 9 m depth. In October 2014, an upper halocline at 2 m depth separated the mixolimnion in two layers consisting of cold, well-oxygenated fresh water at the surface and a layer of warmer, more saline water below, with fluctuating oxygen content.

Hydrography profiles of the lake differed between the two sampling times (Figure 1 and Table S1). In May 2012, the mixolimnion was located closer to the surface at 1–5 m depth, whereas it extended down to 9 m depth in October 2014. The measured oxygen concentrations were also higher overall in the mixolimnion in 2014 than in 2012. In 2012, the lake was anoxic below 5.5 m depth, whereas in 2014 it was only anoxic below 9 m depth. In 2012, the temperature was very similar between the different water layers of the lake with the lowest temperature, 8.9 °C, observed at the surface, and the highest temperature, 10.7 °C, at 7–9 m depth. The salinity profile indicated a gradual increase in salinity from 3 ppt at the surface to 19.9 ppt at 10 m depth, and 20.4 ppt at 15 m depth. In October 2014, a different profile was recorded, and an upper halocline at 2 m depth separated cold, well-oxygenated freshwater from a layer of warmer, more saline water below. Within this water layer, the oxygen content fluctuated between 3 and 1.5 ppm. At 8.5 m depth, we found the main chemocline separating the anoxic monimolimnion from the mixolimnion was located at 9–12 m depth. At this depth, the salinity increased from 17.9 to 20.1 ppt, and the temperature decreased from 16.6 to 12 °C.

### 3.2. Alpha Diversity and Microbial Community Structure

**Figure 2:**
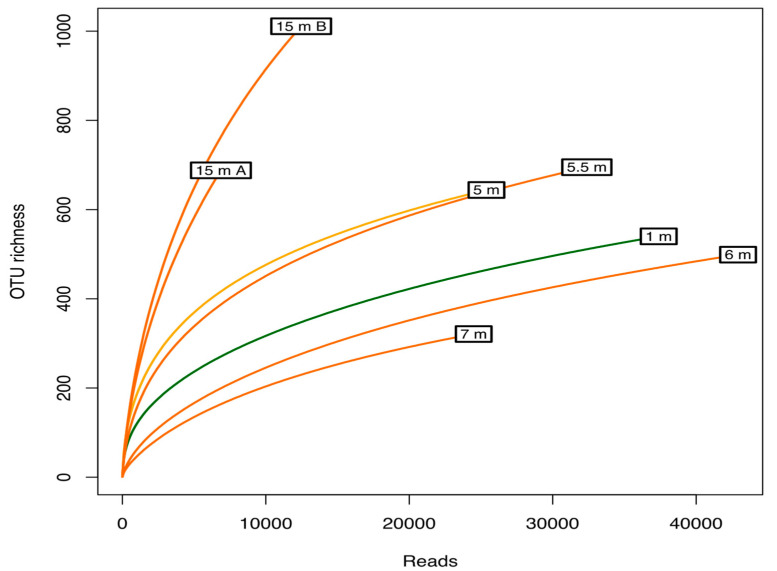
Rarefaction curves for the seven samples showing the diversity detected compared with the predicted total diversity. The *x*-axis represents the number of sequences sampled, while the *y*-axis represents a measure of the species richness detected, estimated with the Chao1 index.

Pyrosequencing of 16S rRNA gene amplicons (metabarcoding) resulted in a total of 181,903 reads after quality filtering and denoising, distributed over 2278 unique OTUs. The deepest sequenced sample (15 mA) yielded 6868 reads. Rarefied richness, calculated as the expected richness at these sequencing depths, ranged from 164 (7 m depth) to 763 (15 m depth). Rarefaction curves indicated that the diversity did not reach saturation in any of the samples, and that the communities at 15 m were more diverse than others (Figure 2). The Chao1 index, a non-parametric estimator of the lower bound of total richness that is less dependent of differences in sequencing depth, also indicated considerably higher richness in the bottom communities compared to other samples (Table 1). Further, the communities at 6 and 7 m depths in the monimolimnion showed a considerably lower Shannon diversity (H’ ≤ 1.39 vs. H’ > 3.39 at other depths) as well as Pielou evenness (J’ < 0.25 at 6–7 m vs. J’ > 0.6 at all other depths), indicating that they were dominated by fewer species and that richness was strongly affected by rare OTUs, possibly including those from dead cells.

**Figure 3:**
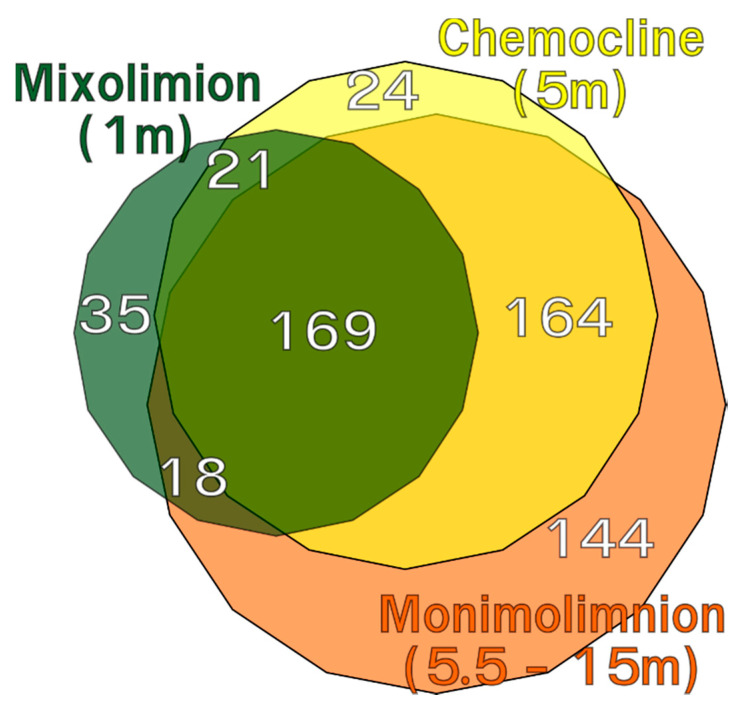
Venn diagram showing the distribution of shared OTUs across the different water layers. White numbers indicate the number of OTUs in each possible subset, adjusted for differences in sequencing depth.

A large core community represented by 169 OTUs (29%) was present in all samples throughout the water column (illustrated as a Venn diagram in Figure 3). Out of the 495 OTUs detected in the monimolimnion (the most diverse water mass), 144 were unique for this water mass, while 164 were shared with the community at the chemocline. Only 35 OTUs were unique to the mixolimnion, and 24 OTUs were unique to the chemocline. Samples from different layers clustered together. 

### 3.3. Taxonomic Profiles Across Depths

**Figure 4:**
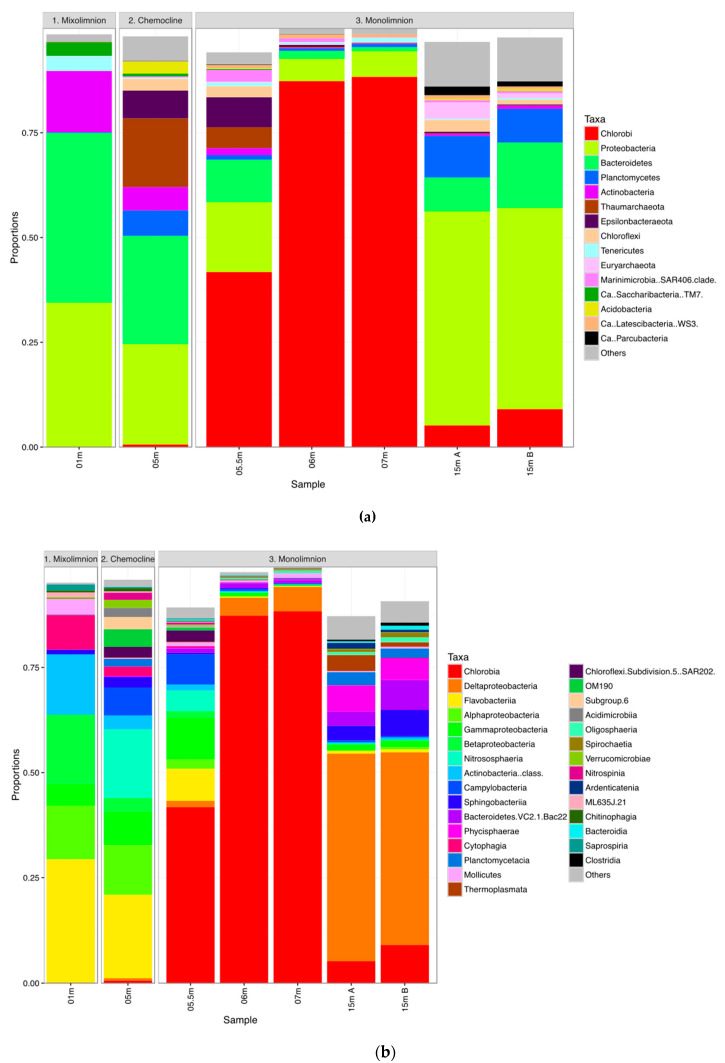
Bacterial community composition in the different water layers in the meromictic lake based on high throughput metabarcoding sequencing of the 16S rRNA gene at (**a**) phylum level and (**b**) class level.

A total of 33 phyla were detected among the 16S rRNA gene sequences throughout the water column, with an average abundance of 0.1% or more (Table S2). In the surface layer (mixolimnion), 90% of the detected microorganisms could be attributed to three phyla: Proteobacteria (35%), *Bacteroidetes* (40%) and Actinobacteria (15%) (Figure 4a). These findings are consistent with other studies suggesting the dominance of these three phyla in freshwater systems [1,31,32]. 

The composition of the mixolimnion was distinctly different from the chemocline and layers deeper down in the water column in the monimolimnion (Figure 4a). At the chemocline (5 m depth), Proteobacteria and *Bacteroidetes* were still predominant, representing 50% of the bacterial community, in addition to Thaumarchaeota (16%), Epsilonbacteraeota (7%), Actinobacteria (6%) and *Planctomycetes* (6%) that became more abundant.

At 5.5 m, just below the chemocline, the phylum Chlorobi dramatically increased, making up 42% of the community, and predominated together with Proteobacteria (17%) and *Bacteroidetes* (10%). A highly distinct shift was also seen at 6 and 7 m depths, where Chlorobi contributed to 87 and 88% of the community, respectively. Interestingly, this group dramatically decreased again with depth and made up less than 9% of the community in the water samples close to the bottom. In these bottom samples, the most predominant phyla were assigned to Proteobacteria (48–51%), along with *Bacteroidetes* (8–16%) and *Planctomycetes* (8–10%) (Figure 4a). 

In all samples, Proteobacteria made up a major fraction of the community. However, there was a distinct shift in their composition, with Alpha- and Betaproteobacteria dominating in the mixolimnion, Alpha- and Gammaproteobacteria dominating the chemocline, and Deltaproteobacteria dominating at 6 m and below, increasing further in the deepest samples close to the bottom where this class reached an abundance of nearly 50%. In these deep samples from the monimolimnion, sulfur-reducing bacteria of the genus *Desulfatiglans* became highly abundant, constituting up to 11% of total reads. Other abundant classes in the mixolimnion were the Phycisphaera (phylum *Planctomycetes*), Sphingobacteria (phylum *Bacteroidetes*) and uncultivated *Bacteroidetes* VC2.1, which all became more abundant in the monimolimnion at 15 m depth (Figure 4b). Around 72% of the sequence reads could not be assigned to a genus or uncultured lineage at genus rank, particularly for the samples closest to the bottom. 

**Figure 5:**
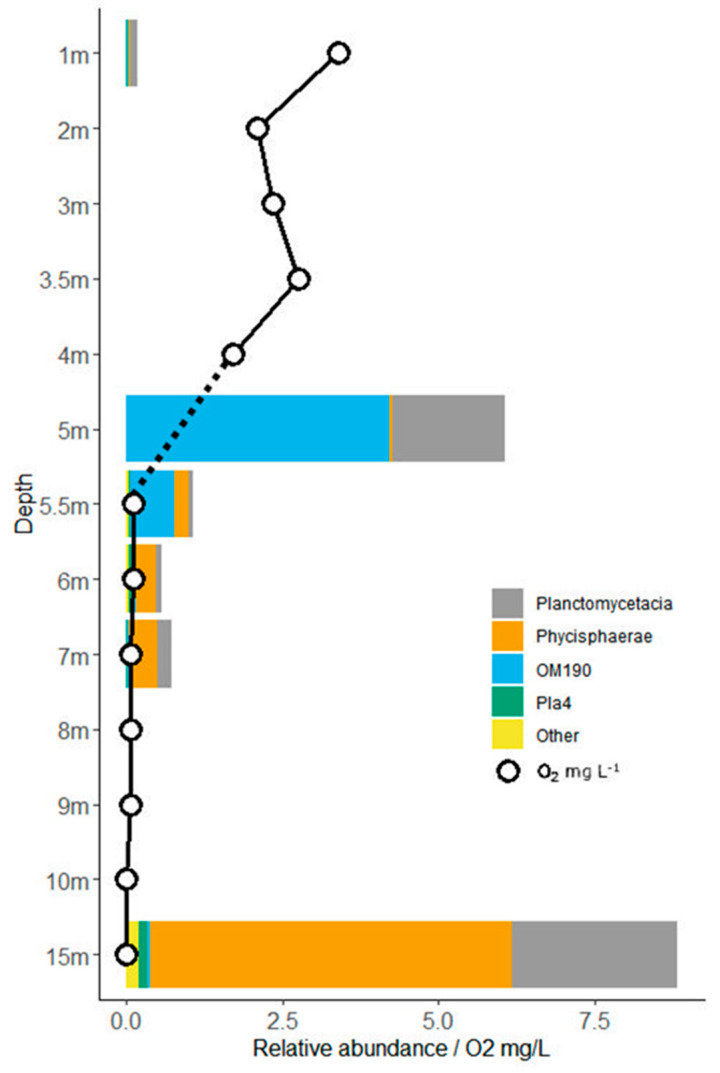
Relative abundance of major *Planctomycete* classes and oxygen (mg/L) concentration in Lake Sælenvannet at selected depths (not to scale) in May 2012. Stippled line indicates that oxygen concentration was not measured at 5 m depth.

In summary, very distinct prokaryotic communities were observed in the different water layers. Also, within *Planctomycetes*, a distinct pattern along the gradient was seen, with a peak of the non-cultivated OM190 group in and right below the chemocline, whereas Planctomycetacia and Phycisphaera dominated in the deep samples (Figure 5).

### 3.4. Planctomycete Distribution along Gradients

*Planctomycete* related sequences were found both in the oxic and anoxic parts of the lake sampled in 2012, but showed an uneven and highly variable distribution throughout the water column, with the highest relative abundances (8–10%) in the saline anoxic monimolimnion layer at 15 m depth (Figure 5). At the chemocline at 5 m depth, 6% were assigned to *Planctomycetes*, whereas the surface water (mixolimnion) and the area just below the chemocline in the monimolimnion consisted of 1% or less (Figure 5). In the chemocline, most *Planctomycete* sequences were affiliated with the uncultivated lineage OM190, which accounted for 4.2% of the bacterial community. The second-largest planctomycetal group, with a relative abundance of 1.8%, was the family Planctomycetaceae in the order Planctomycetales. In the anoxic monimolimnion at 5.5, 6 and 7 m depth, a gradual transition in the most abundant *Planctomycetes* was observed, from OM190 at 5.5 m depth, to Phycisphaerae at 7 m depth (Figure 5). In the monimolimnion at 15 m depth, Phycisphaerae was the most common (3–7% of reads), together with Planctomycetacia (2–3%). The samples closer to the bottom (15 m) also differed significantly in *Planctomycete* diversity from the upper water layers and had a higher number of unique OTUs, especially within the Phycisphaerae (138–168 OTUs at 15 m vs. only 17 OTUs at 1 m, in spite of only being twice as abundant (see Table S2).

**Figure 6:**
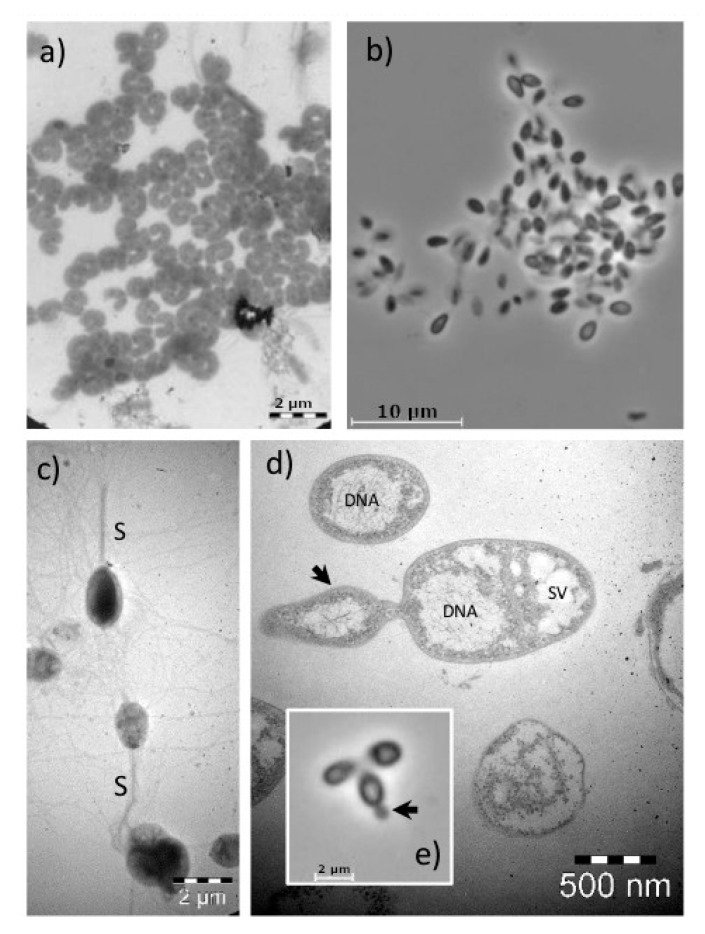
Transmission scanning electron micrographs of the microorganisms from the lake (**a**); Cell morphology characteristics of Chlorobia in water from 6 m depth sampled in 2012 (**b**); Planctomycetacia isolate SV_10m_w forming aggregates (**c**), cells of Planctomycetacia isolate SV_15mW (negative staining) produce long stalks (S); Cells of Planctomycetacia isolate 9mbW (**d**) and Planctomycetacia isolate 7mR (**e**) with budding reproduction. The thin sectioning of the cells in (**d**) shows the densely packed DNA of the cells surrounded by ribosomes and storage vacuole (SV) like structures.

With transmission electron microscopy, a high abundance of magnetotactic bacteria were observed around the chemocline, in addition to *Cholorobium* (Figure 6a). By setting up enrichment cultures aiming for the isolation of *Planctomycetes*, we obtained 10 confirmed isolates from the different layers. Only one of the isolates retrieved in pure cultures was detected in the 454 sequence library.

### 3.5. Planctomycetes Isolates

**Figure 7:**
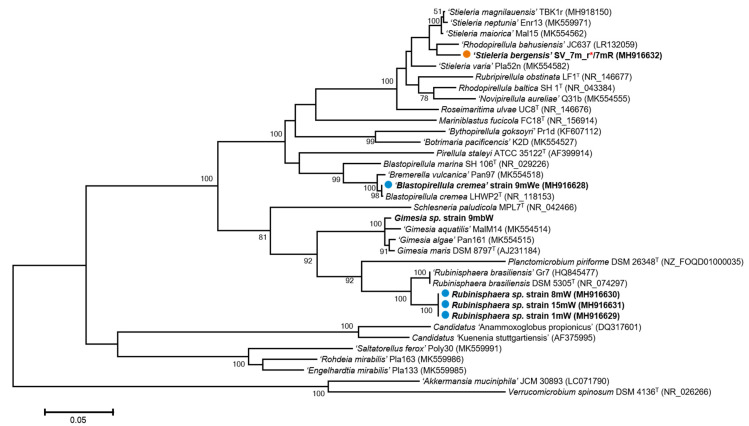
Maximum likelihood tree of isolates and affiliated strains. The evolutionary history was inferred by using the maximum likelihood method based on the Tamura–Nei model. Evolutionary analyses were conducted in MEGA6. Isolates obtained in this study are marked in bold. Strains previously included by Calisto et al. [33], 9mWE, 8mW, 15mW and 1mW, are marked with ●. Strain 7mR was previously included by Calisto et al. and in Wiegand et al. [33,34] and is marked with ●. Red asterisks (*) indicates the name used for the strain [34]. All other strain names are consistent with the name used by Calisto et al. [33] and in NCBI-GenBank. Strains 4mW, 7mW, 8.5mW and 10mW have identical 16S rRNA gene sequences to strains 1mW, 8mW and 15mW, but are not included in the tree.

Only aerobic conditions resulted in successful enrichment. From the water samples collected in 2014, enrichment cultures were initiated in M30 medium and transferred to Gelrite plates containing M13 medium after 7–11 days. Colonies appearing on the plates were inspected with light microscopy, and potential *Planctomycete* isolates were identified on solid Gelrite medium as cells presenting typical plantomycetal-like traits such as rosette formation (Figure 6b), stalks (Figure 6c) and budding reproduction (Figure 6d,e). DNA from potential *Planctomycetes* were extracted, subjected to sequencing and compared with known *Planctomycetes* for confirmation of phylogenetic affiliation. Five of the isolated strains (9mWe, 7mR, 1mW, 8mW and 15mW) have previously been included in a study of secondary metabolite production in *Planctomycetes* [33]. The evolutionary history was inferred by using the maximum likelihood method, based on the Tamura–Nei model. Evolutionary analyses were conducted in MEGA6 (Figure 7). 

Ten novel isolates from four different genera related to the family Planctomycetaceae were obtained in pure culture (Table S2 and Figure 7). Of these, one strain representing a new species within the genus *Stieleria* (strain 7mR) was isolated just above the chemocline at 7 m depth. Strain 7mR was genome sequenced and included in the novel genus *Stieleria* by Wiegand et al. [34], where it was described as “*Stieleria bergensis* SV_7m_r”. Strain 9mWe showed 100% 16S rRNA gene sequence similarity to *Blastopirellula cremea* LHWP2. Both of these were found to have budding reproduction (Figure 6e). No sequences identical to these isolates were found in the 454 library, but seven distantly related OTUs were seen within the *Rhodopirellula* clade. Another isolate obtained at 9 m depth was the strain 9mbW. It showed 99% 16S rRNA gene similarity to *Gimesia maris*, *G. algae* and *G. aquatilis*, and was also identical to one of the OTUs from the 454 library. This isolate also showed budding reproduction, and by TEM, folded membrane structures could be seen, together with open compartments that might be storage vacuoles. Seven isolates (strains 1mW, 4mW, 7mW, 8mW, 8.5mW, 10mW and 15mW) were retrieved from seven different depths in all water layers affiliated with 96% 16S rRNA gene identity to the *Rubinisphaera brasiliensis*. All strains appeared as 1–2 mm white colonies with stalks and budding. Folded internal membrane structures and storage globules were also seen in the TEM images (Figure 6b,c). No sequence similarity to these isolates was seen in the 454 library. 

## 4. Discussion

In correspondence with other meromictic lakes, Sælenvannet possesses a simple food web dominated by planktonic prokaryote and eukaryote microorganisms in the upper aerobic mixolimnion, clearly separated by a transition zone with a distinctly different community composition in and around the chemocline (halo- and oxycline). In the lower monimolimnion, where anaerobic conditions appeared, the observed prokaryote community differed from the one in the upper layers, and sulphate-reducing and sulphur-oxidizing bacteria were predominant. In the aerobic mixolimnion, aerobe heterotrophic organisms dominated. One of these was *Candidatus* Aquiluna, a typical freshwater species within the Actinobacteria. This is a photoheterotrophic organism containing actinorhodopsin which most likely benefits from the high light intensities in the surface layers. Other heterotrophic organisms, such as *Loktanella* sp., *Brevundimonas* and MWH.UniP1 aquatic group were also found to be abundant. 

Within the chemocline where anoxic conditions were established, green sulphur photosynthetic bacteria (*Chlorobium* spp.), which are strict anaerobes, became abundant. These are known from other studies to replace eukaryotic phototrophs like *Euglena* sp. [14,15]. In a metagenomic study of the meromictic Ace Lake in Antarctica by Ng and collaborators from 2010 [35], the functional potential of the green sulphur bacteria dominating the lake indicated that the dominant species, designated C-Ace, possessed chlorosomes with extremely efficient light-capturing capabilities enabling phototrophy and growth potential at very low light intensities. In our study, we found the *Chlorobium* to be highly predominant around the chemocline, where the light conditions were optimal for this functional group, but we also found the *Chlorobium* to be present throughout the water column all the way to the deep samples. These efficient light capture capacities might therefore be the explanation why we observed *Chlorobium* spp. at all examined depths in Lake Sælenvannet. 

Our data further showed that Proteobacteria were a major component of the bacterioplankton at all depths, but there was a distinct shift from Alphaproteobacteria in the mixolimnion (autotrophs and heterotrophs) to Delta-, Zeta- and Gammaproteobacteria in the monimolimnion (chemolithotrophs and sulphate reducers). Further, the Epsilonbacteraeota (formerly “Epsilonproteobacteria”) was more abundant than Proteobacteria at 5 to 5.5 m depth. 

In the anoxic part of Lake Sælenvannet, reductive processes took place. The descriptive sequencing method may distinguish the microorganisms responsible for these processes. It was therefore surprising that in the water masses just beneath the chemocline, chemolithotrophic organisms such as sulphur- and iron oxidizers appeared. For instance, sequences affiliated with the sulphur oxidizing Epsilonbacteraeota family Thiovulaceae, capable of sulphur oxidation, represented 5% of the abundance at this depth and 6% at the mixolimnion. Further, marine neutrophilic chemolithotrophic bacteria able to oxidize ferrous iron into ferric iron, *Mariprofundus* sp. (Zetaproteobacteria), represented 1.1% of the sequence reads at this depth. This indicated that iron is present in this part of the water column together with the previously described high concentrations of sulphur [14]. Sequences affiliated with strict anaerobic purple sulphur bacteria, such as *Thiorhodospira*, were also present below the chemocline. Sulphate reducing bacteria are common in the sulphate rich waters of meromictic lakes where they reduce sulphate to sulphide by means of a number of electron donors, including H_2_, fatty acids, alcohols and aromatic compounds. In the deepest water layers of Lake Sælenvannet, as much as 40% of the sequences were affiliated to Deltaproteobacteria, with the majority of these being potential sulphate reducers (mainly Desulfobacteraceae constituting 29–38% and Desulfarculaceae constituting 7–11%).

Previous studies on meromictic lakes have revealed that both bacteria and archaea show a clear vertical distribution throughout the oxic and anoxic water column and that they play a role in nitrogen cycling [14,36,37]. In this study, we found that the number of bacteria populations dominated, both in the oxic and in the anoxic zones. The archaea were more or less absent from the surface and were found at 6 and 7 m. Interestingly, the archaeal abundance was found to be most predominant at 5 m, constituting 17% of the sequences, then they decreased to 5% at 5.5 m and 3% at the bottom. Most of these archaeal sequences were affiliated to Thaumarchaeota (Ca. *Nitrosopumilales*), an important candidate for the ammonium oxidation process in the lake.

Our study shows a distinct structure within the microbial community throughout the lake that has arisen as the result of distinct physic-chemical parameters and profiles in the lake, defining the individual members of the community. Overall, the phylogenetic analyses show that the prokaryotes dominating the lake are bacteria, with relatively few archaea in the monimolimnion.

Some recent publications have reported, by massive parallel sequencing approach, that *Planctomycetes* represent between 1–22% of the freshwater microbial community [3,38,39]. Thus their presence and functions might be important to elucidate their role in the ecosystem. 

In this study, we enriched specifically for *Planctomycetes* from samples obtained from all layers of the water column and isolated 10 strains from seven different depths into the culture. Two of the isolates (strains 7mR and 9mWe) were affiliated with the genera *Stieleria* and *Blastopirellula*, respectively. Most strains in these clades are characterized as aerobes, with some notable exceptions, such as *Blastopirellula marina*, which can also ferment glucose and reduce nitrate to nitrite [23], and one strain isolated from a sulphur-rich spring which is able to reduce elemental sulfur to sulfide [40]. Isolate 9mWe was retrieved from a 9 m depth from anaerobic water masses. It is closely related to *Blastopirellula marina*, based on its 16S rRNA gene sequence. It is therefore interesting to speculate if they are capable of simply surviving in these water masses, or if they are involved in one or more anaerobic processes. The majority of the isolates (*n* = 7) were affiliated with the genus *Rubinisphaera*, with the closest cultivated isolate being *Rubinisphera brasiliensis*, with 96% similarity on 16S rRNA gene. It is notable that these highly similar isolates were retrieved from seven different depths (1, 4, 7, 8, 8.5, 10 and 15 m), spanning from aerobic to anaerobic water masses, with significantly different salinities and chemical conditions. Interestingly, a highly similar clone sequence (99% identity, Gene Bank id; DQ015774) was obtained from the anoxic layer of the permanently stratified Antarctic Lake Bonney, which display many of the same environmental conditions as Lake Sælenvannet [41]. This could indicate that some species within the *Rubinisphaera* genus are particularly well adapted to the steep environmental gradients that are found in permanently stratified lakes. Members of *Planctomycetia* are generally known for having large genomes [5,34,42], which might indicate large phenotypic plasticity and the ability to quickly adapt to extreme changes in their environment, such as those found in meromictic lakes. The recent study by Wiegand and collaborators [34] contributes substantially to our understanding of this enigmatic *Planctomycete* group of organisms, with 79 functionally described and genome sequenced isolates, including one of the strains isolated in this study, strain 7mR, now named *Stieleria bergensis*.

The 10 isolates that we obtained by cultivation were relatively similar to previously isolated and cultivated *Planctomycetes* from aquatic environments. We also see that there was a large difference in the number of *Planctomycetes* that we could obtain in pure culture compared to what we could find in molecular diversity studies, as only one of our isolates, *Gimesia* sp. strain 9mbW, was also found in the 454 library, pointing out the relevance of bacterial isolation for the comprehension of this phylum. Two interesting results were found in the 454 library. First, the peak of *Planctomycetes* around the chemocline were dominated by the uncultured OM190 clade, with Planctomycetacia dominating, and second was the increase of *Planctomycetes* at the bottom with an increase of Phycisphaerae and Planctomycetacia. The most striking difference was the high abundance of OM190 around the chemocline. In a study by [3], OM190 was detected in half of the lakes studied, indicating that this clade is one of the most common lineages in the oxygenated hypolimnion. OM190 was also found in abundance on the surface of kelp [17] (associated with sulphur-rich carbohydrates) in marine and freshwater sediments [5,30] and associated with particles in marine waters [43,44]. Therefore, this group of *Planctomycetes* might be very well adapted to sulphur-rich environments and an important contributor in the degradation of sulphur compounds and remineralization. Okazaki and colleagues suggested that OM190 might contribute to remineralization in the oxygenated part of the hypolimnion in lake environments [3]. This hypothesis is congruent with our observation of their distribution in Lake Sælenvannet, where they were the most abundant group of *Planctomycetes* in the transition zone between oxic and anoxic conditions (Figure 5).

Additionally, the high abundance of *Phycisphaera* sequences at the bottom in the monimolimnion was interesting, and is consistent with other studies indicating that this group is abundant in freshwater environments [3,5]. Some members of the *Phycisphaera* group are known facultative anaerobes [45,46], and based on their abundance in the anoxic zone in Lake Sælenvannet; we may assume they harbor a similar niche there. Unfortunately, we were not able to obtain any *Phycisphaera* isolates from these samples. 

In this study, it was striking to find the same isolates at several different depths, spanning from aerobic to complete anaerobic conditions. This might be due to a disturbed oxygen profile at the time of sampling, or due to the sinking of organic particles after a bloom. However, the possibility remains that these isolates are capable of living (active or inactive) throughout the water column. Overall, our results contribute to the growing understanding of *Planctomycetes* as ubiquitous and important contributors to ecological processes in aquatic environments and highlights the importance of a continued effort to isolate novel strains.

## 5. Conclusions

Using environmental DNA barcoding, we found that there was a distinct shift in the microbial community composition, although Proteobacteria made up the major fraction of the community (up to 90% in the surface layer). A large core community (29%) was present in all samples throughout the water column. In terms of alpha diversity, rarefaction curves indicated that the diversity did not reach saturation in any of the samples, and the highest diversity was found in the deepest water layers at 15 m depth. *Planctomycetes* were found to vary between 2 and 10% of the community, and a distinct pattern along the gradient was also seen within this phylum, with a peak of the non-cultivated OM190 group in and just below the chemocline. The classes Planctomycetacia and Phycisphaera were found to dominate in the deeper samples. This study resulted in successful isolation in pure culture of ten novel isolates affiliated to four different genera from the family Planctomycetaceae. One strain closely related to *Blastopirellula cremea* was isolated from 9 m depth, and two novel strains affiliated to the genera *Stieleria* and *Gimesia* were isolated at 7 and 9 m depths, respectively. Interestingly, we retrieved seven isolates with identical 16S rRNA gene sequences from seven different depths which varied greatly in salinity and chemical composition. These isolates likely represent new species affiliated to *Rubinisphaera*. The adaptation of novel *Planctomycetes* to water depths spanning the entire chemical gradient could indicate a high phenotypic plasticity and/or a very efficient survival strategy. Overall, our results showed the presence of a diverse group of *Planctomycetes* in Lake Sælenvannet, with a strong potential for novel adaptations to chemical stress factors.

## Figures and Tables

**Table 1:** Diversity statistics.

Dataset	Reads	Singletons	Richness	Rarified Richness	Shannon	Evenness	Chao
Mixo_1m	37,391	148	540	271	3.839	0.610	824
Chemocline_5m	25,382	109	644	416	4.470	0.691	861
Mono_5.5m	32,382	101	695	387	3.391	0.518	988
Mono_8m	42,922	67	499	199	1.379	0.222	774
Mono_7m	24,494	27	321	164	1.390	0.241	522
Mono_15mA	6868	130	688	688	3.951	0.605	1299
Mono_15mB	12,464	271	1011	763	4.252	0.615	1640

## Supplementary Materials

The following are available online at https://www.mdpi.com/2076-2607/8/8/1150/s1, Table S1: Temperature, salinity and oxygen concentrations measured in Lake Sælenvannet in 2012 and 2014, Table S2: Taxonomic_classification.
