# Supplementary material for: Planctomycetes as a Vital Constituent of the Microbial Communities Inhabiting Different Layers of the Meromictic Lake Sælenvannet (Norway)
Source: Microorganisms. 2020 Jul 29;8(8):1150. doi: 10.3390/microorganisms8081150 (PMC7464441; doi:10.3390/microorganisms8081150)
Supplement: Supplementary file 1 [file microorganisms-08-01150-s001.zip › Supplementary Table S1.docx]

Supplementary table 1: Temperature, salinity and oxygen concentrations measured in Lake Sælenvannet in 2012 and 2014.

| **May 2012** | |  |  |
| --- | --- | --- | --- |
| **Depth (m)** | **Temperature (°C)** | **Salinity (ppt)** | **O_2_ (mg L^-1^)** |
| 0 | 8.9 | 3 | 3.5 |
| 1 | 9.1 | 5 | 3.4 |
| 2 | 9.8 | 8.5 | 2.1 |
| 3 | 9.5 | 9.8 | 2.35 |
| 3.5 | 10 | 10.5 | 2.75 |
| 4 | 9.8 | 11.8 | 1.7 |
| 5.5 | 9.6 | 12.5 | 0.1 |
| 6 | 10 | 13.8 | 0.1 |
| 7 | 10.6 | 15.9 | 0.05 |
| 8 | 10.7 | 18 | 0.05 |
| 9 | 10.7 | 19.5 | 0.05 |
| 10 | 10.5 | 19.9 | 0 |
| 15 | 9.5 | 20.4 | 0 |
|  |  |  |  |
| **October 2014** | |  |  |
| **Depth (m)** | **Temperature (°C)** | **Salinity (ppt)** | **O_2_ (mg L^-1^)** |
| 0 | 1 | 8 | 9 |
| 1 | 2 | 11 | 9 |
| 2 | 12.5 | 15.6 | 7 |
| 2.5 | 13.8 | 16.8 | 4 |
| 3 | 14.5 | 17.2 | 3 |
| 3.5 | 15 | 17.4 | 2 |
| 4 | 15.5 | 17.6 | 2.5 |
| 4.5 | 16 | 17.4 | 1.5 |
| 5 | 16.2 | 17.3 | 1.5 |
| 5.5 | 16.5 | 17.2 | 2.7 |
| 6 | 16.8 | 17.1 | 3 |
| 7 | 17 | 17 | 2.7 |
| 8 | 17.3 | 16.8 | 1.5 |
| 9 | 17.5 | 16.6 | 0 |
| 10 | 18 | 16 | 0 |
| 11 | 20 | 14 | 0 |
| 12 | 20.1 | 12 | 0 |
| 15 | 20.2 | 11 | 0 |
